# Supplementary figures and images for: Kininogen-1 as a protein biomarker for schizophrenia through mass spectrometry and genetic association analyses
Source: PeerJ. 2019 Jul 18;7:e7327. doi: 10.7717/peerj.7327 (PMC6642793; doi:10.7717/peerj.7327)

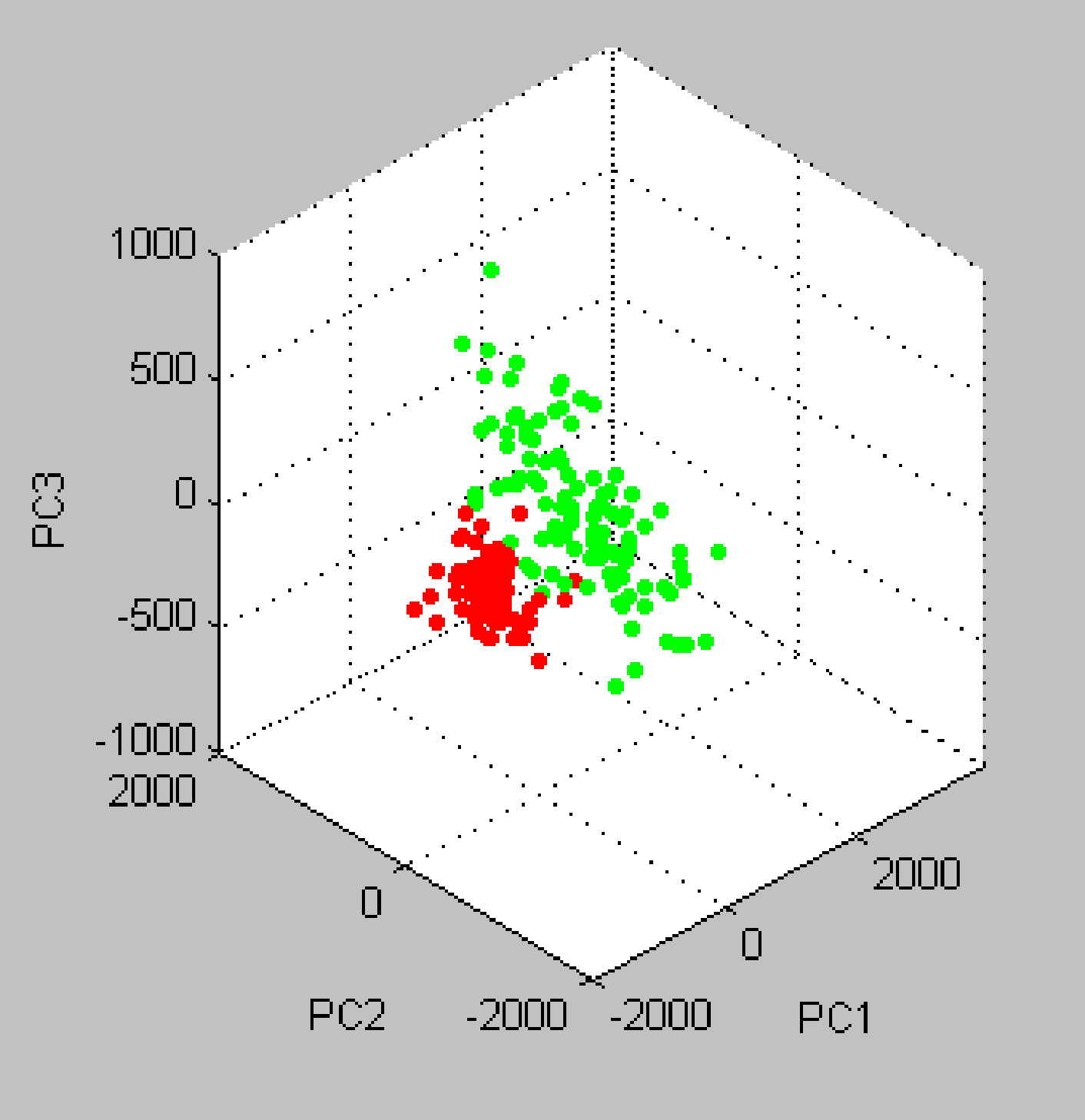

Supplement: Supplemental Information 2 — Red, schizophrenia patients; Green, healthy controls. [file peerj-07-7327-s002.png]
